# LPA-Induced Thromboxane A2-Mediated Vasoconstriction Is Limited to Poly-Unsaturated Molecular Species in Mouse Aortas

**DOI:** 10.3390/ijms25136872

**Published:** 2024-06-22

**Authors:** Krisztina Vén, Balázs Besztercei, Anna Janovicz, Noémi Karsai, Jerold Chun, Gábor Tigyi, Zoltán Benyó, Éva Ruisanchez

**Affiliations:** 1 Institute of Translational Medicine, Semmelweis University, 1085 Budapest, Hungary; egyed.ven.krisztina.eszter@semmelweis.hu (K.V.); besztercei.balazs@semmelweis.hu (B.B.); janovicz.anna@semmelweis.hu (A.J.); noemi.karsai@nottingham.ac.uk (N.K.); gtigyi@uthsc.edu (G.T.); benyo.zoltan@med.semmelweis-univ.hu (Z.B.); 2 Department of Neurology, Semmelweis University, 1085 Budapest, Hungary; 3 HUN-REN-SU Cerebrovascular and Neurocognitive Disorders Research Group, 1094 Budapest, Hungary; 4 Division of Physiology, Pharmacology and Neuroscience, School of Life Sciences, Queen’s Medical Centre, University of Nottingham, Nottingham NG7 2RD, UK; 5 Sanford Burnham Prebys Medical Discovery Institute, La Jolla, CA 92037, USA; 6 Department of Physiology, University of Tennessee Health Sciences Center, Memphis, TN 38163, USA

**Keywords:** lysophosphatidic acid, lysophosphatidic acid receptor 1, thromboxane, vasoconstriction

## Abstract

We have previously reported that, in aortic rings, 18:1 lysophosphatidic acid (LPA) can induce both vasodilation and vasoconstriction depending on the integrity of the endothelium. The predominant molecular species generated in blood serum are poly-unsaturated LPA species, yet the vascular effects of these species are largely unexplored. We aimed to compare the vasoactive effects of seven naturally occurring LPA species in order to elucidate their potential pathophysiological role in vasculopathies. Vascular tone was measured using myography, and thromboxane A_2_ (TXA_2_) release was detected by ELISA in C57Bl/6 mouse aortas. The Ca^2+^-responses to LPA-stimulated primary isolated endothelial cells were measured by Fluo-4 AM imaging. Our results indicate that saturated molecular species of LPA elicit no significant effect on the vascular tone of the aorta. In contrast, all 18 unsaturated carbon-containing (C18) LPAs (18:1, 18:2, 18:3) were effective, with 18:1 LPA being the most potent. However, following inhibition of cyclooxygenase (COX), these LPAs induced similar vasorelaxation, primarily indicating that the vasoconstrictor potency differed among these species. Indeed, C18 LPA evoked a similar Ca^2+^-signal in endothelial cells, whereas in endothelium-denuded aortas, the constrictor activity increased with the level of unsaturation, correlating with TXA_2_ release in intact aortas. COX inhibition abolished TXA_2_ release, and the C18 LPA induced vasoconstriction. In conclusion, polyunsaturated LPA have markedly increased TXA_2_-releasing and vasoconstrictor capacity, implying potential pathophysiological consequences in vasculopathies.

## 1. Introduction

Lysophosphatidic acid (LPA) is a bioactive lysophospholipid mediator found in all eukaryotic tissues and most biological fluids. LPA mediates and modulates several physiological effects ranging from the development of the nervous system to regulation of the immune response and pathophysiological events like neuropathic pain and fibrosis [1,2,3,4]. LPA also controls blood vessels, including regulation of angiogenesis, vascular wall remodeling [5], and blood pressure [6]. LPA refers to a family of lysophospholipids characterized by a glycerol backbone linked to a phosphate group and a fatty acid or fatty alcohol chain. Acyl-LPA contains an ester bond between the glycerol backbone and the fatty acyl chain, whereas in alkyl-LPA, correctly termed as alkyl glycerophosphates (AGP), the fatty alcohol chain is linked via an ether bond. Acyl LPA is more abundant in biological fluids; however, among their receptors, LPAR5 and PPARγ prefer AGP [7]. LPA and AGP species differ in the number of double bonds in the hydrocarbon chain and their regioisomeric configuration. The hydrocarbon chain can be esterified to either the *sn*1 or the *sn*2 carbon of glycerol. Furthermore, the phosphate group is linked to both the *sn*1 and the *sn*2 carbons in cyclic phosphatidic acids [8].

LPA evokes its effects mainly via six G-protein-coupled receptors, LPAR1-6, and the peroxisome proliferator-activated receptor-γ. In our previous studies, we assessed the expression of LPAR1-5 in primary isolated aortic endothelial cells [9] and the expression of LPAR1-6 in the thoracic and abdominal aortic smooth muscle [10]. In the endothelium, the most highly expressed receptor was found to be LPAR4. Transcripts of LPAR1, LPAR2, and LPAR5 were also detected, whereas LPAR3 transcripts were absent. In smooth muscle, notable expression levels of LPAR1, LPAR2, LPAR4, and LPAR6 were observed, with LPAR6 exhibiting the highest expression, consistent with other existing literature [11]. LPAR1 couples to the Gi/o, Gq/11, and G12/13 proteins. The ligand potency of each LPA receptor is distinct for different LPA species, and multiple studies have reported the distinct structure–activity relationship (SAR) of LPA receptors. The ligand preferences of the LPAR1 were previously comprehensively assessed in Sf9 insect cells and in cells individually transfected with LPA receptor subtypes [12,13].

In the circulation, LPA is mainly produced by autotaxin (ATX), an ectoenzyme with lysophospholipase D activity [14,15]. The substrates of ATX are plasma lysophospholipids, most commonly lysophosphatidylcholine [16]. Traditionally, most reports in the literature have investigated 18:1 LPA, even though it is not the most abundant species in biological fluids. In mammalian plasma, the concentration of LPA is less than 50 nM, and the most common molecular species are 18:2 > 20:4 > 16:0 > 18:1 LPA [17]. The combined LPA concentration is elevated to 5> µM in blood serum, and the relative proportion of the LPA species changes to 20:4 > 18:2 > 16:0 > 18:1 > 18:0 [18]. It is noteworthy that the concentration of LPA is elevated in the plasma of patients with acute coronary syndrome [19] and hypertension [20], particularly the levels of 18:2 and 20:4 LPA [21]. In the event of vascular injury or rupture of an atherosclerotic plaque, the ensuing platelet activation leads to localized production of mainly polyunsaturated LPA species (PU-LPA) dominated by 18:2 and 20:4 species [22]

Recently, the actions of LPA in the cardiovascular field have received heightened attention [21,23]. Several studies have suggested that LPA has significant roles in vascular wall remodeling, atherosclerosis, and associated pathologies [19,24,25]. However, the precise mechanism and their prognostic biomarker utility remain unclear. Elevation of the generation of unsaturated LPA in the small intestine correlates with the severity of atherosclerosis in in Ldlr−/− mice fed a high-fat diet. This elevation increases the formation of reactive oxygen species and oxidized phospholipids, subsequently raises levels of bacterial lipopolysaccharides, and results in systemic inflammation [26]. LPA also contributes locally to atherosclerosis by promoting the migration inflammatory cells, cytokine secretion, and macrophage transformation [27]. So far, little is known about the direct effect of naturally occurring LPA species on the release of vasoactive substances and consequent changes in vascular tone. Under experimental conditions, topically administered 18:1 LPA elicits vasoconstriction in the pial arterioles of newborn piglets, which can be blocked by pertussis toxin (PTX) treatment [28]. It has been also shown that 18:1 LPA enhances the intraluminal pressure-induced myogenic constrictor response in skeletal muscle arterioles. This increase in the myogenic response is not seen after inhibition of LPAR1 and 3 [29].

Our research group has recently reported that the 18:1 LPA elicits nitric oxide release via activation of endothelial LPAR1, inducing vasorelaxation [16]. However, when the endothelial layer is damaged, LPAR1 activation induces cyclooxygenase1 (COX-1)-mediated thromboxane A_2_ (TXA_2_) release, leading to vasoconstriction. LPAR1 is expressed in both primary isolated endothelial cells and smooth muscle, although it is not the predominantly expressed LPA receptor subtype in these cell types [10,30]. Both NO and TXA_2_ are crucial players in vascular homeostasis, not only by affecting the vascular tone but also by modulating other aspects of vascular health, like thrombogenicity, expression of adhesion molecules, oxidative stress, proliferation, and inflammation [31,32,33]. By stimulating NO or TXA_2_ production of the vessels, LPAR1 activation is likely to play important roles in vascular health and disease. Notably, not all of the LPAR1 agonists are able to activate both of these pathways. A limitation of our previous study [10] was that the TXA_2_ pathway was activated using a synthetic agonist of the LPAR1-3 (VPC31143) because 18:1 LPA, a potentagonist of the LPAR1, evoked only very weak vasoconstriction. This raises the question of whether the complex vascular response when a mixture of different LPA species activates LPAR1 signaling, as happens naturally in vivo, is not predictable from the structure–activity relationship (SAR) of the individual molecular LPA species on LPAR1 [12,13]. Therefore, in this study, we compared seven of the physiologically most abundant LPA species in blood plasma and serum, assessing their vasoactive properties in the mouse aorta, in order to gain a better understanding of how they individually modulate vascular homeostasis. 

## 2. Results

### 2.1. Vasorelaxation Elicited by Saturated LPA Species

**Figure 1:**
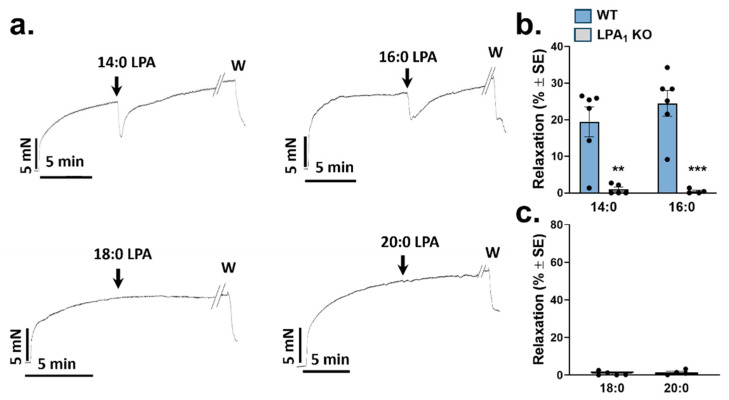
Vasorelaxation evoked by saturated molecular species of LPA. (**a**) Representative recordings of vasorelaxation induced by saturated LPA in PE precontracted thoracic aorta segments prepared from WT mice. Arrows indicate the administration of 10 µM LPA; W, here and in subsequent figures, denotes the washout. (**b**) Vasorelaxation induced by 10 µM 14:0 and 16:0 LPA in aortic rings prepared from WT and *Lpar*1 KO mice (*n* = 6, 6 WT and 5, 4 LPA1 KO; two-way ANOVA; ** *p* < 0.01 vs. WT; *** *p* < 0.001 vs. WT ). (**c**) Vasorelaxation induced by 10 µM 18:0 and 20:0 LPA in aortic segments isolated from WT mice (*n* = 5, 4 unpaired Student’s *t*-test). Bars represent mean ± SEM.

We first examined the activity of the saturated 14:0, 16:0, 18:0, and 20:0 LPA at a concentration of 10 µM in thoracic aorta (TA) segments with intact endothelium. We found that only the LPA 14:0 and 16:0 elicited vasorelaxation (Figure 1a). Vasorelaxation diminished with increases in the length of the fatty acid chain. Next, we tested the action of 14:0 and 16:0 LPA in aortas prepared from *Lpar1* KO mice. As shown in Figure 1b, relaxation in the aortic rings was abolished (*p* = 0.011 and *p* = 0.009, respectively), suggesting that vasorelaxation is mediated via LPAR1. In sharp contrast, the 18:0 and 20:0 LPA species failed to elicit vasorelaxation in WT TA segments (Figure 1c). Saturated LPA species were dissolved in methanol (MeOH), yielding a final concentration in the organ chambers of <1‰. This concentration had no detectable effect on the precontracted aorta segments. 

### 2.2. Vasoconstrictor Activity of Saturated LPA Species

**Figure 2:**
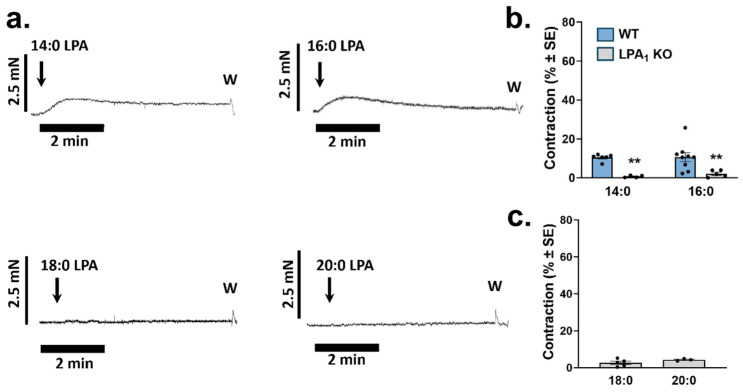
Vasoconstriction evoked by saturated molecular species of LPA. (**a**) Representative recordings of vasoconstriction induced by saturated LPA in endothelium-denuded aortic segments prepared from WT mice. Arrows indicate the administration of 10 µM LPA. (**b**) Vasoconstriction induced by 10 µM 14:0 and 16:0 LPA in endothelium-denuded aortic rings isolated from WT and *Lpar1* KO mice (*n* = 6, 9 for WT and 4, 5 for LPA_1_ KO; two-way ANOVA; ** *p* < 0.01 vs. WT). (**c**) Vasoconstriction induced by 10 µM 18:0 and 20:0 LPA in endothelium-denuded aortic rings isolated from WT mice (*n* = 5, 3; unpaired Student’s *t*-test). Bars represent mean ± SEM.

In our previous study, vasoconstriction mediated via the LPAR1 elicited by 18:1 LPA was significantly higher in the abdominal aorta (AA) segments compared to TA [15]. Therefore, LPA species were applied to the resting tone of endothelium-denuded AA segments to further investigate the SAR of the constrictor response. Similarly to the vasorelaxation, only 14:0 and 16:0 LPA had measurable constrictor effects. We found that the increasing length of the fatty acid chain resulted in a decrease in the constrictor activity (Figure 2a,c). The constrictor effect of the LPA 14:0 and 16:0 was largely absent in vessels prepared from *Lpar1* KO animals (*p* = 0.013 and *p* = 0.021 respectively), indicating that the LPAR1 mediated the 14:0 and 16:0 LPA-induced vasoconstriction (Figure 2b). We were unable to detect vasoconstriction in response to 10 µM by LPA 18:0 and 20:0. Saturated LPA species were dissolved in MeOH.

### 2.3. Vasorelaxation Caused by Unsaturated LPA Species

**Figure 3:**
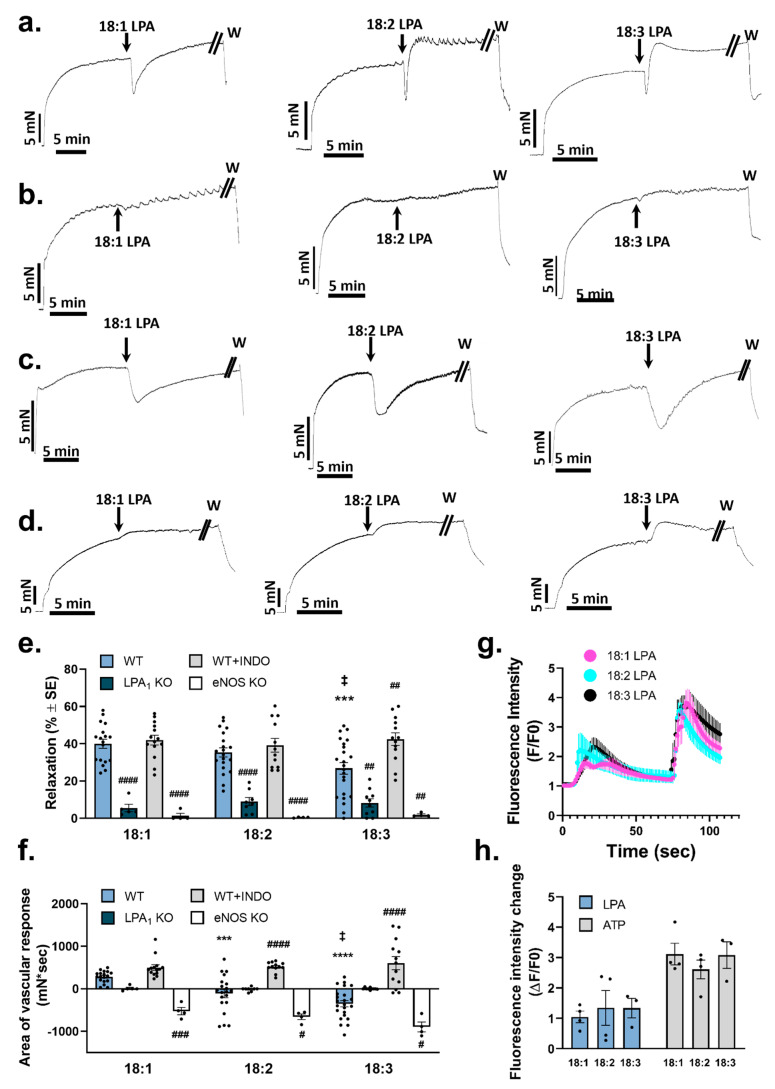
Vasorelaxation induced by unsaturated molecular species of LPA. (**a**) Representative recordings of vasorelaxation elicited by unsaturated LPA in PE precontracted aortic rings isolated from WT mice. Arrows indicate the administration of 10 µM LPA. (**b**) Representative recordings of vasorelaxation elicited by 10 µM of unsaturated LPA in PE-precontracted aortic segments isolated from *Lpar*1 knockout (KO) mice. (**c**) Representative recordings of vasorelaxation elicited by 10 µM of unsaturated LPA in PE-precontracted aortic segments treated with INDO. (**d**) Representative recordings of vasorelaxation elicited by 10 µM of unsaturated LPA in PE-precontracted aortic segments isolated from eNOS KO mice. (**e**) Maximal relaxation activity elicited by 10 µM 18:1, 18:2, and 18:3 LPA in aortic rings isolated from WT, *Lpar1* KO, eNOS KO mice, or WT aortic segments treated with INDO (WT + INDO) [*n* = 18, 21, 25 (WT); 5, 8, 10 (LPA1 KO); 14, 18, 14 (WT + INDO); 4, 4, 4 (eNOS KO) two-way ANOVA; *** *p* < 0.001 vs. 18:1 WT, ‡ *p* < 0.05 vs. 18:2 WT, ## *p* < 0.01 vs. own WT, #### *p* < 0.0001 vs. own WT]. (**f**) Area under the vascular response curve in the first 5 min induced by C18 LPA (10 µM of each) in aortic rings isolated from WT, *Lpar1* KO, eNOS KO mice, or WT aortic segments treated with INDO (WT + INDO) [*n* = 15, 21, 25 (WT); 5, 8, 10 (LPA1 KO); 9, 18, 14 (WT + INDO); *n* = 4, 4, 4 (eNOS KO); two-way ANOVA; *** *p* < 0.001 vs. 18:1 WT, **** *p* < 0.0001 vs. 18:1 WT, ‡ *p* < 0.05 vs. 18:2 WT, # *p* < 0.05 vs. own WT, ### *p* < 0.001 vs. own WT, #### *p* < 0.0001 vs. own WT]. Positive bars indicate dominant relaxation, whereas negative bars indicate a dominant constrictor response. Calculation method for the “area of vascular response” is shown in Supplementary Materials. (**g**) Average trace of fluorescent intensity in Fluo-4 AM-loaded endothelial cells isolated from the aortas of WT mice. Administration of 10 µM of C18 LPA and 10 µM of ATP indicated by arrows (*n* = 4, 4, 3). (**h**) Maximal increase in fluorescent intensity evoked by either 10 µM C18 LPA or ATP (*n* = 4, 4, 3 for LPA; 4, 4, 3 for ATP; one-way ANOVA). Bars represent mean ± SEM.

We evaluated the vasorelaxation elicited by 10 µM concentrations of 18:1, 18:2, and 18:3 LPA. The mono-unsaturated 18:1 LPA showed marked vasorelaxation. In contrast, following the relaxation elicited by PU-LPA, the vascular tone did not return to the precontraction level, but an additional contraction developed (Figure 3a). Comparing the maximal vasorelaxation induced by the C18 LPA species, the relaxation elicited by 18:3 LPA was significantly lower (*p* = 0.002) compared to that of 18:1 LPA (Figure 3e). To obtain better insight into the time course of vasorelaxation, we compared the area of the overall vascular response induced by C18 LPA within 5 min of administration of LPA. This analysis revealed that, as the degree of unsaturation increased, a shift toward vasoconstriction became more apparent (Figure 3f). The evaluation method for the “area of vascular response” is shown in the Supplementary Materials.

To identify the receptor responsible for vasorelaxation, we tested the effect of the unsaturated LPA in aortic segments isolated from *Lpar1* KO mice (Figure 3b). Unsaturated LPA failed to induce a vascular response in vessels prepared from *Lpar1* KO animals, suggesting that these LPAs elicit vasorelaxation via LPAR1 (Figure 3e). We have previously reported that LPAR1 activation can evoke both vasorelaxation and vasoconstriction [9,10]. We hypothesized that, in aortic segments with intact endothelium, the response we observed was the superimposition of these two opposing actions. To separate the relaxation response caused by LPA, it was necessary to inhibit the constrictor component of the response. Based on our previous research, where the LPAR1 activation caused COX-mediated vasoconstriction [10], we applied the C18 LPA to vessels pretreated with 10 µM indomethacin (INDO). By comparing of the peak vasorelaxation induced by these LPA, we found that only vasorelaxation induced by 18:3 LPA increased significantly (*p* = 0.001) after the inhibition of COX (Figure 3e). However, when analyzing the area of the vascular response induced by C18 species of LPA, the relaxation elicited by PU-LPA species increased significantly in the presence of INDO (*p* < 0.0001 for 18:2 LPA; *p* < 0.0001 for 18:3 LPA, Figure 3f). In vessels treated with INDO, no significant difference was detected in the relaxation caused by any C18 LPA species tested (Figure 3c,e,f). Similar responses were detected in mice lacking the thromboxane prostanoid receptor (TP KO, Supplementary Figure S1; details regarding the animals are given in Supplementary Materials). This indicated that acyl-chain-dependent differences in activity might be related to differences in the constrictor efficacy of the C18 LPA. In aortic segments prepared from eNOS KO mice, the LPA-induced vasorelaxation by C18 species was completely diminished (Figure 3d,e), indicating that the relaxation effect was mediated via eNOS signaling by all C18 LPAs examined. In the representative recordings (Figure 3d), C18 LPA-induced vasoconstriction became apparent in these aortas, with an increasing tendency that paralleled the degree of unsaturation. Accordingly, when analyzing the area of the vascular response, all C18 LPA showed a dominant constrictor effect in aortic segments isolated from eNOS KO animals (Figure 3f).

To gain a better understanding of the molecular mechanism of the relaxation activity of C18 LPA species, we measured the Ca^2+^-signals evoked in cultured primary endothelial cells. Consistent with the physiological relaxation response, endothelial cells showed marked elevation in intracellular Ca^2+^ when stimulated by 10 µM of the C18 LPA species, which was independent of the degree of unsaturation.

### 2.4. Vasoconstrictor Activity of Unsaturated LPA

**Figure 4:**
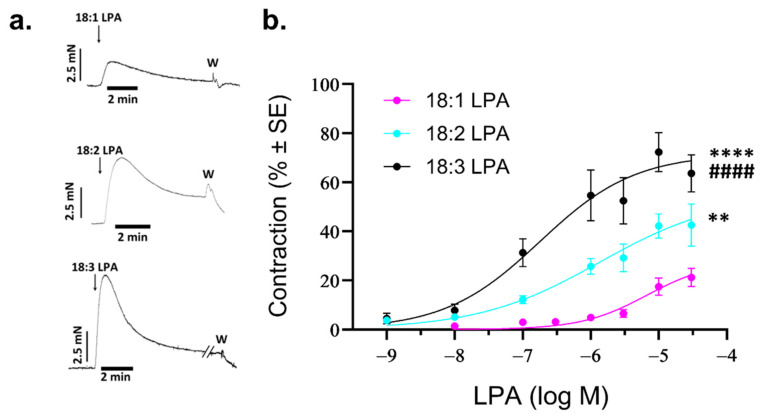
Vasoconstriction evoked by unsaturated molecular species of LPA. (**a**) Representative recordings of vasoconstriction induced by 10 µM of unsaturated LPA in endothelium-denuded AA rings prepared from WT mice. Arrows indicate the administration of 10 µM LPA. (**b**) Dose–response curves for 18:1, 18:2, and 18:3 LPA-induced vasoconstriction in endothelium-denuded AA rings isolated from WT mice (*n* = 3–25; two-way ANOVA; ** *p* < 0.01 vs. 18:1 LPA, **** *p* < 0.0001 vs. 18:1 LPA, #### *p* < 0.0001 vs. 18:2 LPA). Bars represent mean ± SEM.

Vasoconstriction induced by 10 µM of LPA 18:1, 18:2, and 18:3 (Figure 4a) showed an increasing trend that paralleled the higher number of double bonds. Considering the pathophysiological importance of unsaturated LPA species, we analyzed the dose–response relationship of their vasoconstrictor activity. Analysis of the dose–response relationship showed that 18:1 LPA is a weak constrictor (EC_50_: 6.67 µM, E_max_: 27.9%), whereas the efficacy and potency of the constrictor activity of PU-LPA increased significantly (18:1 vs. 18:2 LPA *p* = 0.0012; 18:1 vs. 18:3 LPA *p* < 0.0001; 18:2 vs. 18:3 LPA *p* < 0.0001) [18:2 LPA: EC_50_: 1.35 µM, E_max_: 57.8%; 18:3 LPA: EC_50_: 0.18 µM, E_max_: 71.6%] (Figure 4b).

### 2.5. Signaling Mechanism of Vasoconstriction Induced by Unsaturated LPA

**Figure 5:**
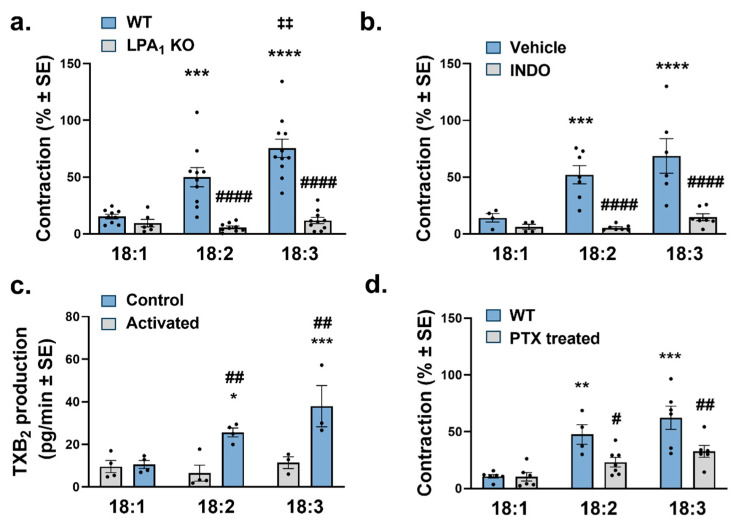
Signaling mechanisms underlying the constrictor activity of unsaturated LPA species. (**a**) Vasoconstriction elicited by LPA species 18:1, 18:2, and 18:3 (10 µM of each) in endothelium-denuded AA segments isolated from WT and *Lpar*1 KO mice [*n* = 9, 10, 11 for WT and 6, 9, 10 for LPA1 KO; two-way ANOVA; *** *p* < 0.001 vs. 18:1 WT, **** *p* < 0.0001 vs. 18:1 WT, ‡‡ *p* < 0.01 vs. 18:2 WT, #### *p* < 0.0001 vs. own WT]. (**b**) Vasoconstriction induced by C18 LPA (10 µM of each) in control (Vehicle) and cyclooxygenase-inhibited (INDO) endothelium-denuded vessels isolated from WT mice [*n* = 4, 7, 6 for vehicle and 4, 7, 7 for Indo; two-way ANOVA; *** *p* < 0.001 vs. 18:1 WT, **** *p* < 0.0001 vs. 18:1 WT, #### *p* < 0.0001 vs. own WT]. (**c**) TXB_2_ production in aortas before (Control) and after (Activated) treatment with 10 µM of C18 LPA (*n* = 4, 4, 3 for control and 4, 4, 3 for activated vessels; two-way ANOVA; * *p* < 0.05 vs. 18:1 Control, *** *p* < 0.001 vs. 18:1 control, ## *p* < 0.01 vs. own control] (**d**) Vasoconstriction elicited by C18 LPA (10 µM of LPA 18:1, 18:2 and 18:3) in endothelium-denuded aortic rings isolated from mice treated with vehicle (WT) or pertussis toxin (PTX-treated)) [*n* = 6, 4, 6 for WT and 6, 7, 6 for PTX treated; two-way ANOVA; ** *p* < 0.01 vs. 18:1 WT, *** *p* < 0.001 vs. 18:1 WT, # *p* < 0.05 vs. own WT, ## *p* < 0.01 vs. own WT]. Bars represent mean ± SEM.

To establish the role of LPAR1, we examined the constrictor effect of C18 unsaturated LPA species in aortic segments isolated from *Lpar1* KO mice. Vasoconstriction induced by C18 unsaturated LPA was completely abolished in vessels prepared from *Lpar1* KO mice, suggesting that the constrictor effect was elicited via LPAR1 (Figure 5a). Similarly, after inhibiting COX with indomethacin, vasoconstriction evoked by C18 unsaturated LPA completely disappeared (Figure 5b). As COX activation leads to the release of the potent vasoconstrictor thromboxane (TX), we measured the TXB_2_ released in the effluent of vessels exposed to 10 µM of each C18 unsaturated LPA. PU-LPA 18:2 and 18:3, but not 18:1 LPA, increased TXB_2_ production in the aortic segments, suggesting a role of prostanoids responsible for the constrictor effect (Figure 5c). Because COX activation is coupled to the G_i_ protein signaling pathway, next, we investigated the constrictor activity of C18 unsaturated LPA in mice treated intraperitoneally with PTX for 5 days. In aortas isolated from these mice, the average contraction induced by 18:2 and 18:3 LPA was significantly reduced compared to vessels from vehicle-treated mice (Figure 5d).

### 2.6. Vasoconstriction Caused by a Combination of LPA Species in a Ratio That Emulates Its Distribution in Plasma

**Figure 6:**
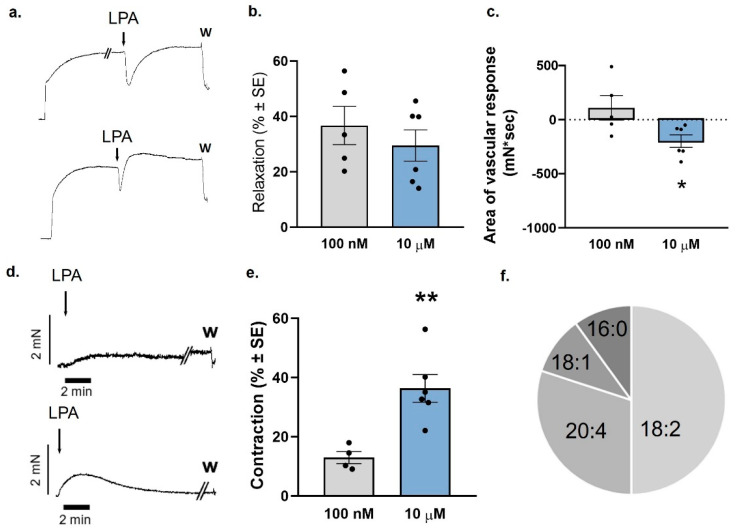
Vasorelaxation and vasoconstriction evoked by a combination of LPA species with a mixture resembling that of plasma. (**a**) Representative recordings of the vascular effect induced by 100 nM (top) and 10 µM (bottom) of the combination of LPA species in intact TA rings prepared from WT mice. Arrows indicate the administration of the combination; W stands for wash out. (**b**) Evaluation of the amplitude of vasorelaxation of the LPA mixture [*n* = 5 for 100 nM, 7 for 10 µM]. (**c**) Area of vascular response of the LPA mixture [*n* = 5 for 100 nM, 7 for 10 µM; unpaired *t*-test, * *p* < 0.05 vs. 100 nM]. (**d**) Representative recordings of vasoconstriction induced by 100 nm (top) and 10 µM (bottom) of the combination of LPA species in endothelium-denuded AA rings prepared from WT mice. Arrows indicate the administration of the combination; W stands for washing out. (**e**) Vasoconstriction induced by 100 nM and 10 µM of the combination of LPA species in endothelium-denuded AA rings isolated from WT mice [*n* = 4 for 100 nM, 6 for 10 µM; unpaired *t*-test, ** *p* < 0.01 vs. 100 nM]. Bars represent mean ± SEM. (**f**) Relative abundance of the individual LPA molecular species, represented in a pie chart.

Based on data from the literature [17], we combined LPA species in a ratio that best resembled that of the ratio of LPA species found in plasma at a quasi-physiological concentration of 100 nM and examined its relaxing effect on TA segments with intact endothelium (Figure 6a) and its constrictor effect on endothelium-denuded AA segments, respectively (Figure 6d). Interestingly, the amplitude of the relaxing effect was the same in both concentrations examined (Figure 6b). However, at higher pharmacological concentrations of 10 µM, the relaxation became shorter and turned into a constriction, as shown by a significantly more negative area of vascular response (Figure 6c). As illustrated in Figure 6, the combination of LPA species had a minor constrictor effect at a concentration of 100 nM, which was similar to that of LPA concentrations found in plasma (Figure 6d,e). However, when the mixture reached a concentration of 10 µM, it induced a marked vasoconstriction (Figure 6d,e). This concentration of total LPA mimics what can be reached locally in the vicinity of a ruptured atherosclerotic plaque.

## 3. Discussion

Herein, we report for the first time that naturally occurring molecular species of LPA elicit markedly different vasorelaxation and vasoconstriction, which parallels their different potencies in releasing TXA_2_. These differences underscore the importance of the fact that elevated plasma levels of specific LPA species exert vastly different cardiovascular effects. It is well documented that serum and plasma levels of LPA molecular species are differentially and significantly increased in certain pathophysiological conditions; however, until now, we have found no experimental evidence for the differential actions of individual naturally occurring LPA species on vascular tone.

Our results indicate that the vasoactive effects triggered by the examined LPA species are solely mediated through activation of the LPAR1, as the LPA species failed to induce vasorelaxation or vasoconstriction in vessels obtained from *Lpar1* KO mice. 

Saturated LPAs with shorter chains have weak vasorelaxant and constrictor effects, but as the hydrocarbon chain becomes longer, these effects disappear. The 14:0 LPA was found to have the capability to induce both vasorelaxation and vasoconstriction in aortic rings, which aligns with the findings reported by Bandoh et al., who demonstrated that 14:0 LPA can activate signaling pathways through the LPAR1, albeit to a lesser extent [12].

Unsaturated LPAs have higher potency in terms of evoking both vasorelaxation and vasoconstriction via LPAR1. This agrees with the literature data, as the structure–activity relationship of the LPAR1 in transfected rat hepatoma model cells shows the following rank order: 18:2 > 18:3 > 20:4 > 18:1 > 16:0 > 18:0 [13]. Regarding the endothelial effects of the investigated PU-LPA, the vasorelaxant activity decreases with the number of double bonds. The difference in the induced vasorelaxation was even greater when the area of the overall response was analyzed, although this could also be attributed to different rates of elimination. We reported previously that LPAR1 activation-induced vasorelaxation is completely mediated via eNOS activation and intracellular Ca^2+^-release, as it is lacking in eNOS KO vessels [9]. In this work, we showed that eNOS mediates the relaxing effect of the PU-LPA as well, indicating that no other vasodilatory mechanism is initiated by LPAR1 activation. However, the difference in the relaxant activity of the C18 LPA seems not to be due to their different potencies to induce NO, but rather due to the counteracting vasoconstrictor activity elicited only by PU-LPA. This is supported by their ability to evoke similar vasorelaxation when the counteracting vasoconstriction is inhibited by indomethacin. Furthermore, they evoke similar Ca^2+^-signals in primary endothelial cells. 

We found that unsaturated LPA species elicit vasoconstriction via COX activation and TXA_2_ production in the aorta. The constrictor response correlates with the released TXA_2_, which parallels the increasing number of double bonds up to 3, the highest we have tested. Inhibition of COX diminished the vasoconstriction induced by the C18 LPA, whereas PTX treatment of the mice significantly inhibited the constrictor response. Intravenously injected LPA species induce transient hypertension in mice [6]. Although unsaturated LPAs evoke hypertension more efficiently than saturated LPAs, which is in agreement with our findings, this hypertensive response is mediated primarily by LPAR4 and LPAR6 [6]. Relaxation and constriction of the aorta are not major influencing factors on blood pressure. However, our findings pertain to thrombogenicity at the vessel wall because certain LPAs are more prone to eliciting NO release, whereas others are capable of considerable TXA_2_ release. In the case of 18:1 LPA, aside from its ability to induce vasorelaxation, the released NO may further inhibit thrombo- and atherogenesis by preventing platelet activation, aggregation, and adhesion, and via inhibition of smooth muscle cell proliferation and superoxide formation [34]. However, under pathophysiological conditions due to platelet activation, the more potent PU-LPA species, which are the dominant species in plasma, release TXA_2_ that can overwrite the relaxation effect of NO. This is even more pronounced when the endothelial layer is damaged, leading to vasoconstriction, increased thrombogenicity, and vascular wall remodeling [30]. Clearly, further research is needed to clarify whether these vascular effects are also present in smaller conductance arteries, especially the epicardial coronary arteries or carotid artery, as LPA-evoked vasoconstriction may lead to decreased tissue perfusion in the heart and brain, which is pronounced in cases of endothelial injury. Interestingly, 18:1 LPA can elicit vasorelaxation, but fails to evoke TXA_2_ release and produces modest vasoconstriction, though both effects are mediated by the LPAR1. This virtual contradiction can be reconciled by the different pharmacological and signaling properties of the LPAR1 in different cell types of the vascular wall. Because vasorelaxation is elicited by G_q_, whereas vasoconstriction is evoked by the G_i_ signaling pathway, another possible explanation is a signaling bias of the LPAR1, which has been already reported in human lung fibroblasts [35]. Regarding the vasoactive effects, signaling bias can be due to differences either in G_q_ and G_i_ signaling or in β-arrestin and G protein coupling. Another possible explanation may involve tissue-specific expression of the lipid phosphate phosphatases, which are responsible for the degradation of the LPA [36], and their different ligand preferences may explain the different constrictor and relaxant effects of the 18:1 LPA. The combined effects of LPA species in a mixture with a plasma-like composition, dominated by polyunsaturated species of LPA, elicited marked vasoconstriction at high, yet pathologically relevant, concentrations of 10 µM.

It is important to mention, however, that in this study, we did not examine the possible effects of physiological determinants (e.g., age, sex) on the vasoactive responses evoked by LPA; instead, we focused on describing the underlying signaling pathways. Therefore, further investigation is required to include both sexes and different age groups in order to gain a better understanding of the LPA-induced vasoactive effects in mouse aortic segments.

**Figure 7:**
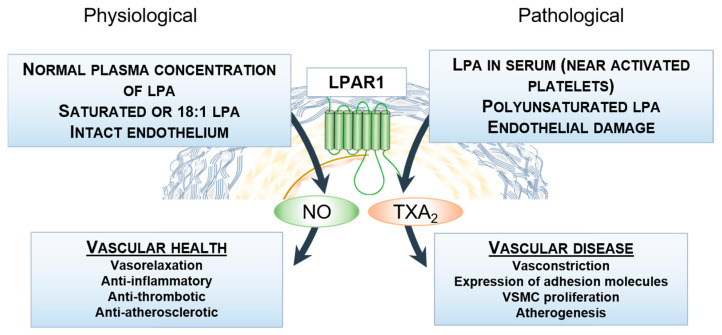
Proposed role of naturally occurring LPA species in vascular health and disease.

In conclusion, polyunsaturated C18 LPA species induce marked vasoconstriction via COX-1-derived TXA_2_ release, which overrides their endothelial NO-mediated vasorelaxant effect. This phenomenon might become particularly important in cases involving endothelial injury and subsequent platelet activation, where the consequentially released PU-LPA can contribute to the progression of vasospasms and enhance platelet activation via initiating thromboxane-mediated signaling. Our results provide an opportunity to advance the understanding of the vasoactive effects of naturally occurring LPA species under both physiological and pathophysiological conditions (Figure 7). We note that, for in vitro and in vivo studies of LPA-elicited cellular and tissue responses, inclusion of different molecular species of LPA will required in future studies.

## 4. Materials and Methods

### 4.1. Animals

Wild-type (WT) C57Bl/6J mice (ages 32–38 weeks) and eNOS-knockout (eNOS KO) mice of C57BL/6J genetic background were purchased from Charles River Laboratories (Isaszeg, Hungary). *Lpar1* knockout (LPAR1 KO) mice were provided by Jerold Chun (Scripps Research Institute, La Jolla, CA, USA), and have been maintained after backcrossing to C57BL/6 since 2008. In Figure 1, Figure 2, Figure 3 and Figure 5, the WT group represents a pooled cohort of C57Bl/6J and LPAR1 littermate control mice, as their responses to LPA were similar (Supplementary Figures S2 and S3). All animals were housed at a constant temperature with a 12/12 h light/dark cycle in a pathogen-free, humidity-controlled environment with ad libitum access to standard laboratory chow and water. To determine the participation of G_i_ protein signaling of LPA-induced vasoconstriction, mice were injected with PTX (source) intraperitoneally for 5 days at a dose of 30 μg/kg body weight to inhibit the heterotrimeric G_i_ protein. Control mice were injected with 20% glycerol in saline, which was used to dissolve PTX. The inhibitory effect of PTX was monitored by the disappearance of the acetylcholine (ACh)-induced decrease in heart rate (Figure S4). 

### 4.2. Reagents and Chemicals

The saturated LPAs (14:0, 16:0, 18:0 LPA) were purchased from Avanti Polar Lipids (Alabaster, AL, USA), whereas the 20:0 LPA and unsaturated LPA (18:1, 18:2, 18:3, 20:4 LPA) were obtained from Echelon Biosciences Inc. (Salt Lake City, UT, USA). Saturated LPAs were dissolved in MeOH, and unsaturated LPAs were dissolved in physiological saline solution and sonicated immediately before administration. All other reagents were obtained from either Thermo Fisher Scientific (Waltham, MA, USA) or Sigma-Aldrich (Hamburg, Germany).

### 4.3. Preparation of Vessels

Adult male mice were transcardially perfused with 10 mL heparinized (10 IU/mL) Krebs solution (119 mM NaCl, 4.7 mM KCl, 1.2 mM KH_2_PO_4_, 2.5 mM CaCl_2_, 1.2 mM MgSO_4_, 20 mM NaHCO_3_, 0.03 mM EDTA, and 10 mM glucose at 37 °C, pH 7.4) after ether-induced deep anesthesia. To measure vasorelaxation, thoracic aortic segments (TA) [9] were used (Supplementary Figure S5), whereas for the testing of vasoconstriction, abdominal aortic segments (AA) were isolated [10]. Fat and connective tissue were removed from the vessels under a dissection microscope. The cleaned aortas were cut into 3 mm long segments and placed into the chamber of the wire myograph, which was filled with 6 mL aerated (95% O_2_–5% CO_2_), 37 °C warm Krebs solution. In the case of measuring vasoconstriction, the endothelial layer was removed by pulling a 3/0 surgical thread through the lumen of the vessel. The absence of the endothelium was confirmed by the disappearance of the Ach-evoked vasorelaxation.

### 4.4. Measurement of the Vascular Tone

Aortic segments were pulled on stainless steel vessel holders (200 mm in diameter) of the wire myograph (610M Multiwire Myograph System; Danish Myo Technology A/S, Aarhus, Denmark). The segments were incubated for 30 min, while the resting tone of the vessels was adjusted as previously determined [37]. As the first step of our protocol, Krebs solution was replaced by 124 mM K^+^ Krebs (made by isomolar replacement of Na to K) solution for 1 min, and the chambers were flushed thoroughly several times with Krebs until the vessels reached their resting tone again. To induce vasoconstriction, 10 μM PE was administered to the chamber, followed by 0.1 μM ACh to test the reactivity of the smooth muscle and endothelial cells, respectively. After rinsing with Krebs solution several times, vessels were exposed to 124 mM K^+^ Krebs for 3 min, which served as a reference contraction throughout this study. After re-establishment of the resting level of the vascular tone by washing the chambers with fresh Krebs solution, the cumulative dose–response relationships of PE (10 nM–10 μM) and ACh (1 nM–10 μM) was examined, respectively. In the case of measuring vasorelaxation, if the Ach-elicited relaxation was less than 60% of the PE-induced pre-contraction, the vessel was excluded from the experiment because of the presumed endothelial damage. When examining vasorelaxation, vessels were precontracted to 80–100% of the reference contraction by an appropriate concentration of PE, and, after contraction had stabilized, the effects of LPA were determined. When measuring vasoconstriction, LPA was administered to vessels at their resting tone. In dose–response experiments on LPA-induced vasoconstriction, it was administered in single doses to avoid homologous desensitization of the LPAR1 [38]. At the end of the experiment, to test the robustness of the vessel’s response, 124 mM K^+^ Krebs solution was applied to the resting tone for 3 min. In some experiments, to inhibit COX, vessels were incubated with 10 μM INDO for 20 min prior to the administration of LPA. After rinsing with fresh Krebs, 10 μM INDO was applied again just before LPA administration.

### 4.5. Isolation and Culture of Aortic Endothelial Cells

Complete media for cell cultures was prepared by combining Dulbecco’s Modified Eagle Medium (DMEM) with 10% fetal bovine serum (FBS) and 1% penicillin/streptomycin. The adventitia was removed by gently separating the layers, and TAs were cut into 1–2 mm long segments. These segments were dissociated enzymatically in digestion solution of 1 mL Hank’s Balanced Salt Solution with calcium and magnesium (HBSS++), containing 1/10 solution of 2.5 mg/mL Liberase TM and 30 U/mL Hyaluronidase, and placed into the CO_2_ incubator for 1 h. After incubation, 3 mL of complete medium was added to the mix to stop digestion. The cell suspension was centrifuged at 300× *g* at room temperature for 5 min, and the cell pellet was resuspended in 3 mL of complete medium and recentrifuged. The cells were resuspended in 6 mL pre-warmed complete medium, and one mL was placed into a 6-well plate containing gelatin-coated coverslips 25 mm in diameter. Cells were grown in a humidified incubator (37 °C, 5% CO_2_) for 3 days. 

### 4.6. Intracellular Ca^2+^ Measurement in Primary Aortic Endothelium Cells

After 5–8 days, the isolated cells were washed with HBSS++ three times. The cell-loading solution consisted of 2 μM Fluo-4 AM supplemented with Pluronic F-127 (0.02% *v*/*v*) diluted in HBSS++. Cells were incubated in the cell-loading solution at room temperature for 45 min. After incubation, cells were washed with HBSS++ three times. To allow for AM-deesterification, cells were incubated for another 30 min in HBSS++ at room temperature. Coverslips were placed into the Attofluor cell chamber (Thermo Fisher Scientific) and imaged using a Nikon Ti2-E inverted fluorescence microscope under a 20×/0.75 NA objective. Emission and excitation were recorded at 490 nm and 520 nm, respectively. Images were taken every second, and 10 μM LPA was added after 60 s of baseline measurement. At the end of the experiment, 10 μM of adenosine triphosphate (ATP) was administered to the cells.

### 4.7. Image Analysis

Images were analyzed using the NIS Elements AR 5.21.03. software (Nikon, Tokyo, Japan). After removing the background fluorescence, cells were chosen based on endothelial phenotype, which was validated by the previously described immunofluorescence staining (Figure S6). Relative fluorescence intensities were expressed as ΔF/F_0_, where F is the fluorescence intensity and F_0_ is the mean basal fluorescence intensity. Data were plotted over time, where the *y* axis is F/F_0_ and *x* axis is time in seconds.

### 4.8. Measurement of the TXA_2_ Release

LPA-induced TXA_2_ release was measured by a TXB_2_ enzyme immunoassay kit (Cayman Chemical Co., Ann Arbor, MI, USA). TXA_2_ is thermally unstable; it isomerizes almost immediately into the stable TXB_2_, which was detected in this immunoassay. TAs were isolated as described above. Vessels were cut into 3 mm long segments and incubated in 37 °C HBSS for 1 h. Segments were exposed to 200 μL of warm (37 °C) Krebs solution and gently shaken using an orbital shaker for two min. The supernatant was collected, snap-frozen in liquid nitrogen, and stored at −80 °C. This served as a baseline level of TXA_2_ production of the vessels. Subsequently, 200 μL of 10 μM LPA-containing Krebs solution was added to the segments for 2 min, and the collected supernatant was snap-frozen in liquid nitrogen and stored at −80 °C. The immunoassay was performed according to the instructions of the manufacturer, and TXB_2_ release was expressed as pg/min.

### 4.9. Statistical Evaluation

The Biopac MP100 system and AcqKnowledge 3.9.1.6 software (Biopac System Inc., Goleta, CA, USA) were used to detect changes in the vascular tone. Values of contraction were expressed as percentages of the maximal contraction elicited by 124 mM K^+^-Krebs, whereas relaxation was normalized to the PE-induced precontraction. In the case of vasorelaxation, the overall area of the vascular response was calculated in the following way: the area under the curve was determined over a 5 min period after LPA administration and subtracted from the area given by the precontraction force extrapolated to 5 min (for details and a figure explaining the calculation, see Supplementary Materials). In all experiments, n indicates the number of animals. Statistical analysis was performed with GraphPad Prism software (v.8.01; GraphPad Software Inc., La Jolla, CA, USA). Means and standard errors of the mean (SEMs) are presented on the charts. Outliers were filtered using ROUT (robust regression and outlier removal, ROUT coefficient Q = 1%), and normal distributions were checked by the Shapiro–Wilk test. Student’s unpaired *t*-test was used to compare two variables, whereas for comparing more than two experimental groups, ANOVA was performed, followed by Tukey’s or Sidak’s post hoc test. A *p*-value of <0.05 was considered statistically significant.

## 5. Conclusions

Although the involvement of LPA has been extensively studied in relation to various cardiovascular diseases, including atherosclerosis and hypertension, no previous study has compared the effects of naturally occurring LPA molecular species on vascular tone in situ. Here, we show that saturated LPA and unsaturated LPA species have different actions on the aortic wall. Saturated species have no or minor effects on the vascular tone, whereas unsaturated LPAs have both relaxing and constrictor activities. PU-LPA induced enhanced TXA_2_ production and led to pronounced vasoconstriction. As our experiments were conducted on mouse aortas, it is important to emphasize that these results may not be applicable to resistance vessels that are involved in controlling blood pressure, but due to the dominant increase in PU-LPA species after the activation of blood clotting, this can have far-reaching pathophysiological consequences in cardiovascular diseases.

## Data Availability

The data presented in this study are available upon request from the corresponding author (accurately indicate status).

## Supplementary Materials

The following supporting information can be downloaded at: https://www.mdpi.com/article/10.3390/ijms25136872/s1.
