# Supplementary material for: LPA-Induced Thromboxane A2-Mediated Vasoconstriction Is Limited to Poly-Unsaturated Molecular Species in Mouse Aortas
Source: Int J Mol Sci. 2024 Jun 22;25(13):6872. doi: 10.3390/ijms25136872 (PMC11241118; doi:10.3390/ijms25136872)
Supplement: Supplementary file 1 [file ijms-25-06872-s001.zip › supplementary_materials.pdf]

## SUPPLEMENTARY MATERIALS AND METHODS

### 1. Assessment of the inhibitory effect of PTX in Langendorff-perfused hearts

After mice were anesthetized by intraperitoneal injection of 40 mg/kg pentobarbital (Euthasol 40%; Produlab Pharma BV, Raamsdonksveer, The Netherlands), their hearts were removed. Subsequently, the isolated hearts were cannulated and a modified Krebs–Henseleit buffer (118 mM NaCl, 4.3 mM KCl, 25 mM NaHCO3, 1.2 mM MgSO4, 1.2 mM KH2PO4, 0.5 mM NaEDTA, 2.0 mM CaCl2, 11 mM glucose, 5 mM pyruvate (pH 7.4)) was used for perfusion at a constant, 80 mmHg pressure by a gravitational Langendorff apparatus (Experimetria Ltd., Budapest, Hungary). Following the cannulation of the isolated heart, a 30-min equilibration period was allowed. After the establishment of baseline, 10 μM ACh was infused for 2 min, to assess the presence of the Gi inhibition evoked by the PTX. Haemosys software (Experimetria Ltd., Budapest, Hungary) was used for data acquisition and analysis. Maximal decrease in HR is expressed as a percentage of the baseline.

### 2. Immunofluorescence staining

After measuring the intracellular $Ca^{2+}$-signals, endothelial cells were characterized by their morphological features and cell-specific marker. Cells were washed with Phosphate Buffered Saline (PBS) and fixed with 4% paraformaldehyde at room temperature for 15 min and washed again with PBS. They were permeabilized with 0.25 % Triton X-100 containing PBS (PBST) for 10 min, then washed 3 times with fresh PBS for 5 min. Subsequently, cells were blocked with 1% bovine serum albumin (BSA) in PBS for 1 h, then incubated with primary antibody against CD31 [1:200 dilution; Thermo Fisher Scientific (Waltham, MA, USA)] in 1% BSA-PBST solution at 4°C overnight. On the following day, cells were incubated with goat anti-rat Alexa-Fluor-488 conjugated secondary antibody [(1:500 dilution, Thermo Fisher Scientific (Waltham, MA, USA)] for 1 h at room temperature. Hoechst (1 μg/ml) was used for nuclear counterstaining.

### 3. Evaluation method for the 'area of vascular response'.

The red area is taken by the Biopac AcqKnowledge 3.7.3 software as 'Integral' of the curve in the first 300 s after LPA administration. The blue striped area is calculated as follows: value of the precontraction (mN) x 300 (s). The columns in Figures 3f. and 6c. of the manuscript can be interpreted as follows: the more positive is the 'area of vascular response', the more pronounced (greater and/or longer) is the vasorelaxation; the more negative is the 'area of vascular response', the more pronounced (greater and/or longer) is the vasoconstriction during the first 5 minutes of the effect. A value of zero can mean no effect at all as well as relaxation and constriction of the same magnitude is present.

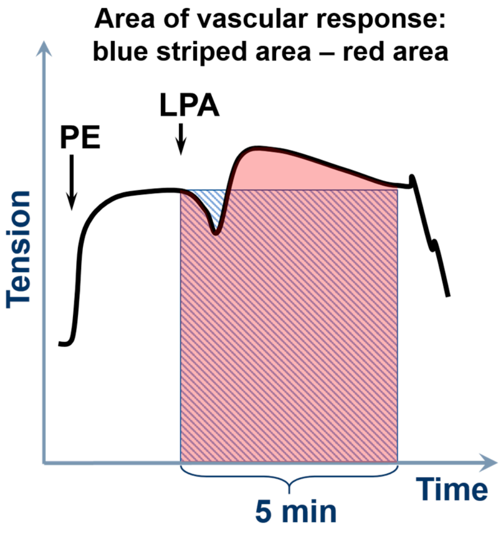


**4. Animals**

TP receptor knock out (TPKO) mice (ages 32-38 weeks) were generously given by Dr. Shuh Narumiyah (Kyoto University, Kyoto, Japan) and have been maintained at the Semmelweis University after backcrossing to C57Bl6 at least 10 times.

## SUPPLEMENTARY RESULTS

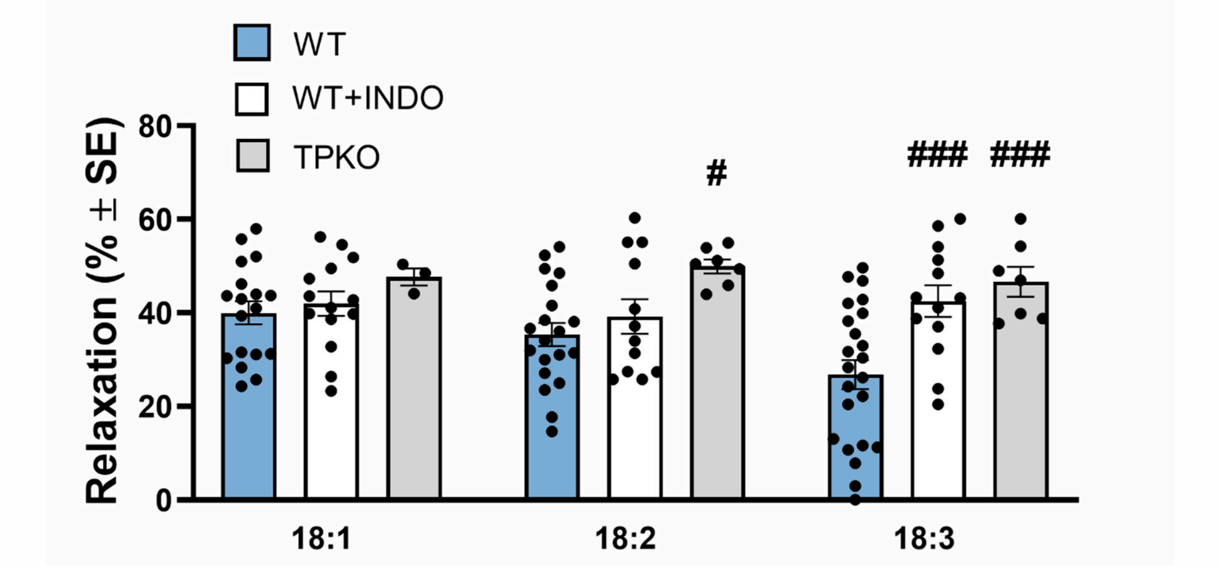


**Figure S1.** Vasorelaxing effect of 18:1, 18:2 and 18:3 LPA in TA segments with intact endothelium WT, TP knock out (TPKO) mice or in TA isolated from WT mice treated with indomethacin (WT+INDO) [n= 18, 20, 23 (WT); 14, 12, 13 (WT+ INDO); 3, 7, 7 (TPKO); two-way ANOVA, ##p <0.01 vs. own WT, ###p <0.001 vs. own WT].

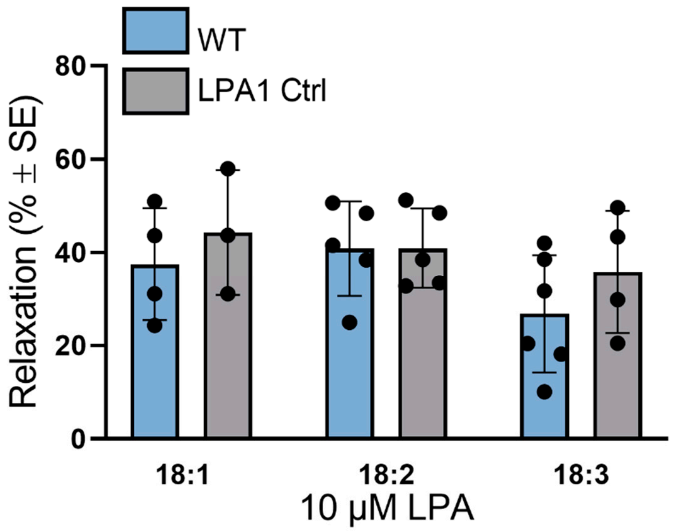


**Figure S2.** Vasorelaxation induced by unsaturated molecular species of LPA in aortas prepared from WT or LPA1 Ctrl mice [n= 4 ,5 ,6 (WT); 3 ,5 ,4 (LPA1 Ctrl); n represent the number of animals, two-way ANOVA]

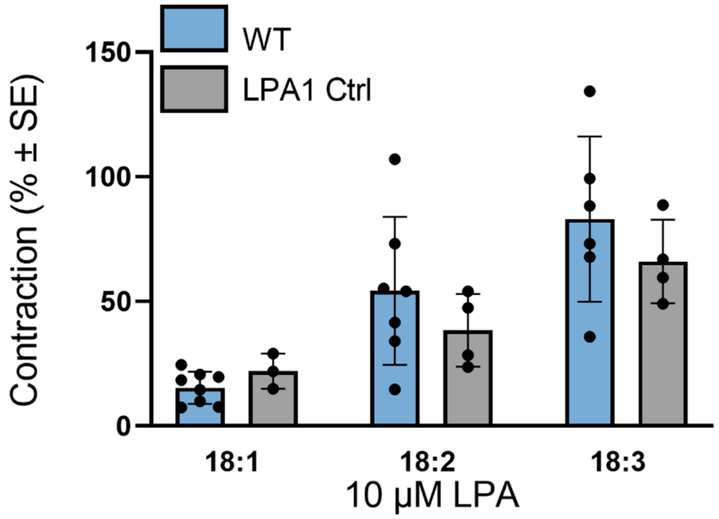


**Figure S3.** Vasoconstriction induced by unsaturated molecular species of LPA in aortas prepared from WT or LPA1 Ctrl mice [n= 8, 7, 6 (WT); 3 ,4 ,4 (LPA1 Ctrl); n represent the number of animals, two-way ANOVA]

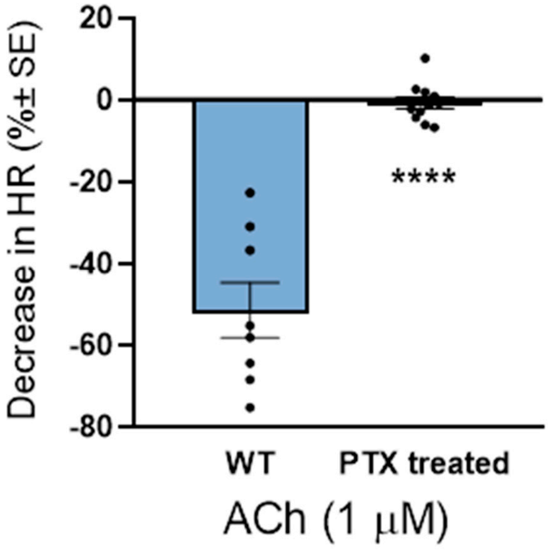


**Figure S4.** Assessment of the inhibitory effect of PTX in Langendorff-perfused hearts. Acetylcholine induced decrease of HR in hearts isolated from WT mice treated with vehicle (WT) or PTX (1 µM of ACh; n= 8, 11; unpaired Student's t-test; ****p <0.0001 vs. WT). Bars represent mean ± SEM.

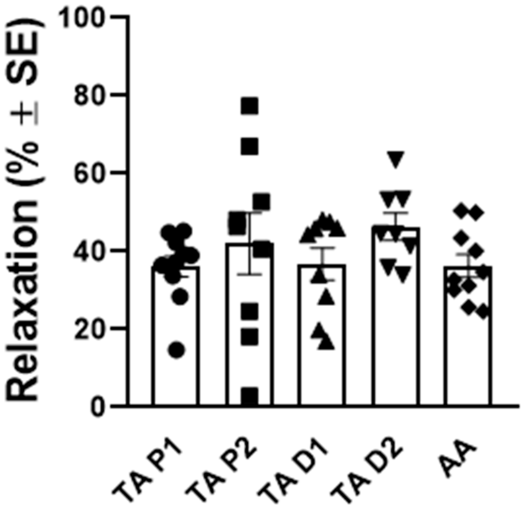


**Figure S5.** Relaxing effect of 18:1 LPA is similar in the different parts of the mouse aorta. P1=first proximal, P2=second proximal, D1=first distal, D=second distal segment of the TA. n = 11, 9, 9, 8, 10. Bars represent mean ± SEM.

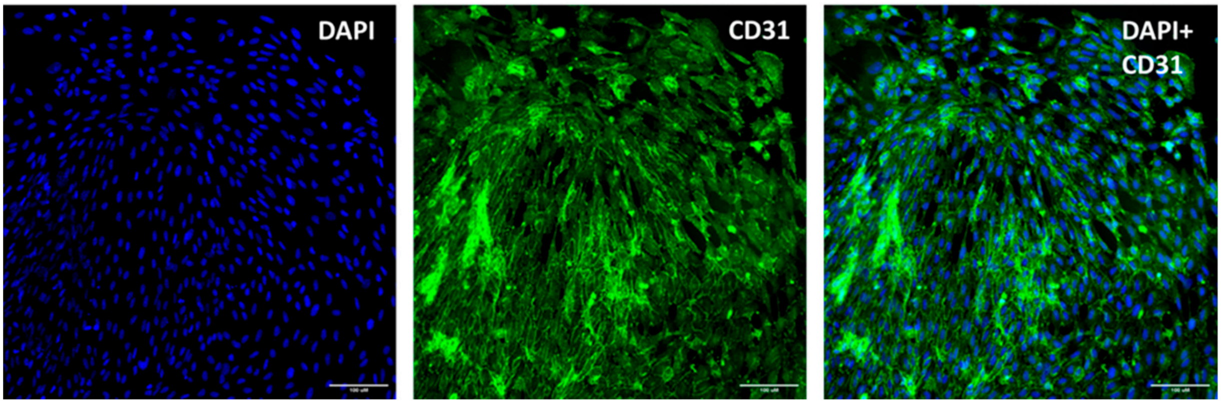


**Figure S6.** CD31 expression in primary isolated vascular cell culture. Immunofluorescence staining with Hoechst (blue) and CD31 (green) confirmed the endothelial origin of the primary cell culture; scale bar: 100 µm.
